# Optical and Microdialysis Monitoring of Succinate Prodrug Treatment in a Rotenone-Induced Model of Mitochondrial Dysfunction in Swine

**DOI:** 10.3390/metabo16010065

**Published:** 2026-01-11

**Authors:** Alistair Lewis, Rodrigo M. Forti, Tiffany S. Ko, Eskil Elmér, Meagan J. McManus, Arjun G. Yodh, Todd J. Kilbaugh, Wesley B. Baker

**Affiliations:** 1 Department of Chemistry, University of Pennsylvania, Philadelphia, PA 19104, USA; alilewis@sas.upenn.edu; 2 Division of Neurology, Department of Pediatrics, Children’s Hospital of Philadelphia, Philadelphia, PA 19104, USA; menezesfor@chop.edu; 3 Resuscitation Science Center, Department of Anesthesiology and Critical Care Medicine, Children’s Hospital of Philadelphia, Philadelphia, PA 19104, USA; kotiff@chop.edu (T.S.K.); mcmanusm@chop.edu (M.J.M.); kilbaugh@chop.edu (T.J.K.); 4 Mitochondrial Medicine and Department of Clinical Sciences, Lund University, 221 00 Lund, Sweden; eskil.elmer@med.lu.se; 5 Abliva AB, 223 63 Lund, Sweden; 6 Department of Physics, University of Pennsylvania, Philadelphia, PA 19104, USA; yodh@physics.upenn.edu

**Keywords:** primary mitochondrial disease, cerebral oxygen metabolism, cytochrome-c-oxidase, diffuse optical spectroscopy, complex I dysfunction

## Abstract

Background/Objectives: Mitochondrial dysfunction is a major cause of brain injury in patients with primary mitochondrial disease. New mitochondrial therapeutics and non-invasive tools for efficacy monitoring are urgently needed. To these ends, succinate prodrug NV354 (methyl 3-[(2-acetylaminoethylthio)carbonyl]propionate) and diffuse optical techniques are promising. In this proof-of-concept study, we characterize NV354’s effects on microdialysis metrics of cerebral metabolism in a swine model of mitochondrial dysfunction and assess the associations of diffuse optical metrics with mitochondrial dysfunction and metabolic improvement. Methods: One-month-old swine received a four-hour co-infusion of rotenone with either the succinate prodrug NV354 (*n* = 5) or placebo (*n* = 5). Rotenone is a mitochondrial complex I inhibitor. Before and during co-infusion, cerebral metabolism was probed with microdialysis and diffuse optics. Microdialysis acquired interstitial lactate and pyruvate levels invasively, while diffuse optics measured changes in oxygen extraction fraction (OEF) and oxidized cytochrome-c-oxidase concentration (oxCCO). Results: Interstitial lactate continually increased in the placebo group (*p* < 0.01), but lactate levels plateaued in the NV354 group (*p* = 0.90). oxCCO also increased in the placebo group (*p* = 0.05), but OEF remained constant (*p* = 0.80). In the NV354 group, oxCCO increased (*p* < 0.01) while OEF decreased (*p* < 0.01). Conclusions: Microdialysis results suggest that NV354 treatment can increase oxygen metabolism in large animals with mitochondrial dysfunction. The optical oxCCO metric was also sensitive to metabolic changes induced by rotenone and NV354 administration.

## 1. Introduction

Patients with primary mitochondrial disease are a heterogeneous population that experience mitochondrial dysfunction due to mutations in nuclear or mitochondrial DNA [1,2,3]. Mitochondrial dysfunction negatively affects almost every organ in the body, but the brain, with its high energy demand and critical dependence on mitochondrial bioenergetics, is especially at risk [2,3,4]. Among the most common causes of mitochondrial dysfunction in these patients is complex I dysfunction [1,5]. Current therapies are mostly limited to symptom management, but new emerging therapies, such as prodrugs and gene therapy, show promise [6,7]. Non-invasive tools that detect metabolic improvements could aid in the precision application of these and other neuroprotective therapies.

Diffuse optical methods hold promise to address this need via their assessment of cerebral oxygen metabolism [8,9,10,11,12]. Here, assessments include a mitochondrial metric that probes the change in redox state of cytochrome-c-oxidase (CCO, also known as complex IV) [13,14,15,16,17,18], and hemodynamic metrics that probe cerebral blood flow (CBF), oxygen extraction fraction (OEF), and an index of cerebral metabolic rate of oxygen derived from CBF and OEF [11,12,19]. In healthy mitochondria, the electron transport chain transfers electrons from complexes I and II to coenzyme Q. Subsequently, electrons are transported through complex III and complex IV (CCO), culminating in the reduction of O_2_ to water. Primary mitochondrial dysfunction impedes the electron in-flow rate to CCO. We hypothesize that this alters the equilibrium redox ratio of CCO, i.e., it decreases the reduced form of CCO (redCCO) and increases the oxidized form (oxCCO). Primary mitochondrial dysfunction is also expected to induce secondary decreases in oxygen metabolism, which will decrease the tissue oxygen extraction from the blood, i.e., OEF decreases. Herein, we aim to show the sensitivity of the optical metrics to complex I dysfunction induced by rotenone in swine. Rotenone is a highly specific complex I inhibitor [20], and its administration in swine was previously shown to increase blood lactate and venous oxygen tension [21].

We also aim to characterize the metabolic effects of the succinate prodrug NV354 (methyl 3-[(2-acetylaminoethylthio)carbonyl]propionate) in the swine model. NV354 is designed to increase the concentration of succinate in mitochondria [22]. The provision of substrate directly to complex II enables increased electron flow through complex II to compensate for impaired electron flow through complex I [22,23]. Succinate itself will not passively transport across cell membranes due to its charged nature. NV354, however, is a stable, water-soluble thioester that does pass through cell membranes. Once inside the cell, NV354 is hydrolyzed by cellular esterases to release succinate (see Supplementary Material), which then enters mitochondria via the dicarboxylate carrier. NV354 has been shown to mitigate acute mitochondrial dysfunction in cellular and rodent models [22,23,24,25,26,27,28,29], but its effects in large animal models have not been studied.

Herein, we used diffuse optical methods together with cerebral microdialysis to monitor cerebral metabolism in a rotenone-induced swine model of complex I dysfunction. Cerebral microdialysis invasively measures the concentrations of interstitial lactate and pyruvate; increases in interstitial lactate and lactate–pyruvate ratio (LPR) are often indicative of decreases in oxygen metabolism [30,31]. We tracked the temporal changes of the optical and microdialysis metrics induced by rotenone poisoning with and without NV354 treatment. We also directly compared mitochondrial and vascular-based optical metabolism metrics during these processes, i.e., we determined the association between the variations of oxidized CCO (oxCCO) and OEF. These proof-of-concept results motivate future research into succinate supplementation in large animal models of complex I dysfunction. To our knowledge, this study also presents the first in vivo optical measurements and comparison of oxCCO and OEF changes induced by primary mitochondrial dysfunction and mitochondrial-targeted drugs.

## 2. Materials and Methods

All animal care and procedures were conducted in adherence to the National Institute of Health Guide for the Care and Use of Laboratory Animals and the European Treaty Series—No. 123 European Convention for the Protection of Vertebrate Animals used for Experimental and Other Scientific Purposes. Approval of all procedures was obtained from the Institutional Animal Care and Use Committee of the University of Pennsylvania. This study is reported in accordance with ARRIVE guidelines.

### 2.1. Study Design

**Figure 1:**
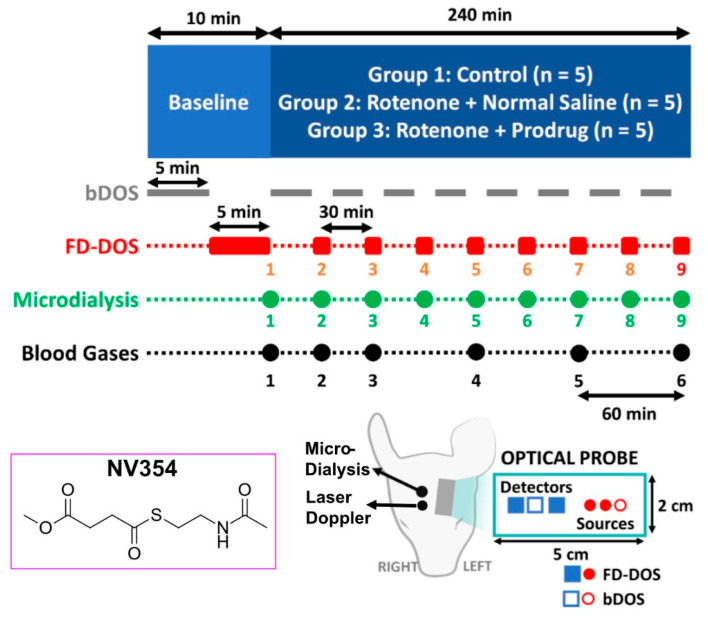
Schematic of study timeline. After baseline measurements (i.e., 5 min bDOS, then 5 min FD-DOS), physiological monitoring was performed for a four-hour IV infusion period (infusion administered according to group). Monitoring comprised 28 min of bDOS (solid gray line) interleaved with 2 min of FD-DOS (red rectangles), cerebral microdialysis sampling (green circles), and systemic blood gas sampling (black circles); continuous cortical perfusion monitoring was also acquired invasively with a laser Doppler probe secured to the frontal dura matter. The FD-DOS/bDOS optical probe was sutured to the skin on the left forehead, and the microdialysis sensor was inserted through a burr hole into the interstitial space of the right frontal cortex. The optical probe comprised one bDOS and four FD-DOS source-detector pairs (bDOS source-detector separation, 3.0 cm; FD-DOS source-detector separations, 1.5, 2.0, 2.5, 3.0 cm). The structural formula of the NV354 succinate prodrug is also depicted.

Non-invasive broadband (bDOS) and frequency-domain (FD-DOS) diffuse optical spectroscopy, and invasive cerebral microdialysis sampling, were performed on 15 one-month-old Yorkshire swine (*Sus scrofa domesticus*, mean [range] weight = 10.8 [9.1–13.2] kg) divided into three groups: a control group (*n* = 5), a rotenone + placebo group (*n* = 5), and a rotenone + prodrug group (*n* = 5). The microdialysis technique provided our primary assessment of brain metabolism. Relative cerebral blood flow was also monitored invasively with a laser Doppler probe (PeriFlux, Perimed Inc., Stockholm, Sweden). Neuromonitoring was performed during a 10 min baseline period and for four hours during intravenous infusions (Figure 1). The rotenone + placebo group received intravenous infusions of rotenone (0.125 mg/kg/h; Sigma-Aldrich, Burlington, MA, USA) and normal saline; the rotenone + prodrug group received intravenous infusions of rotenone (0.125 mg/kg/h) and succinate prodrug (100 mg/kg/h, NV354; Abliva AB, Lund, Sweden); the control group received intravenous infusion of normal saline. The only exclusion criterion for animals during the experiment, established *a priori*, was early euthanasia prior to the end of the 4 h intravenous infusion due to hemodynamic instability. Swine were allocated to each group using block randomization. The researchers present during the experiment were not blinded, but analysis of the microdialysis samples was blinded.

The rotenone + prodrug co-infusion models scenarios wherein intervention occurs during acute metabolic crisis. Thus, this study is designed to provide a proof-of-concept of NV354 prodrug’s ability to maintain mitochondrial function under stress; this maintenance of mitochondrial function is essential to show before testing rescue therapy protocols. Note, based on prior swine work [21], we expected our choice of the rotenone infusion dose to result in complex I dysfunction without hemodynamic instability. Note also, the NV354 infusion dose we used is about twice as large as the doses that resulted in therapeutic benefits in rodent models [23,28,29]. Given the species differences in metabolism and the need to overcome potential blood–brain barrier limitations, this larger dose helps ensure that therapeutic NV354 levels accumulate in the swine brain. Additional pre-study pilot testing of two one-month-old Yorkshire swine demonstrated that administration of this dose over 4 h did not cause hemodynamic instability.

Finally, we note that the neuroprotective benefits of NV354 were observed at 3 h after initiation of treatment in rodent models [28,29]. This observation, along with unpublished data in rats that shows rapid uptake and release of succinate in the brain tissue at 5 min after intravenous bolus injection of ^13^C-labeled NV354 (20 mg/kg), suggests that 4 h of intravenous infusion enables assessment of drug effects after steady-state tissue levels of NV354 are achieved.

Neuromonitoring devices were removed after the monitoring period, but intravenous infusions continued and animals were transferred to a scanner for magnetic resonance spectroscopy (MRS) measurements. After MRS, animals were euthanized via bolus injection of potassium chloride. The total duration of IV infusion prior to euthanasia was approximately 6 h. Our focus herein is on the changes in cerebral physiology measured during the first four hours of infusion (MRS is discussed in Supplementary Material).

### 2.2. Animal Preparation, Anesthesia and Respiratory Management

Swine were initially sedated with intramuscular injections of ketamine (20 mg/kg) and buprenorphine (0.02 mg/kg), followed by 2–3% inhaled isoflurane. After confirmation of adequate sedation via the absence of withdrawal response on toe pinch, swine were intubated with a 32 Fr/CH left-endotracheal tube (Covidien, Medtronic, Dublin, Ireland), and then mechanically ventilated with 2–3% isoflurane and 21% FiO_2_ (positive end-expiratory pressure, 5 cmH_2_O; peak inspiratory pressure, 1–1.5 cmH_2_O/kg) for the duration of the study. The respiratory rate and maximum ventilation pressure were adjusted to maintain the end-tidal CO_2_ between 38 and 42 mmHg. Catheters were placed with ultrasound guidance in the right femoral artery and bilateral femoral veins for arterial blood pressure monitoring, blood gas sampling, and drug administration. A central venous catheter was also placed in the superior vena cava for rotenone infusion; succinate prodrug and normal saline infusions were administered via the femoral vein. Next, neuromonitoring was placed, a hybrid bDOS/FD-DOS optical probe was sutured to the left forehead, a microdialysis catheter (CMA 71 Elite, mDialysis, Stockholm, Sweden) was inserted through a cranial burr hole to a depth of 1–1.5 cm into the brain parenchyma of the right frontal cortex, and a laser Doppler probe was inserted through a cranial burr hole and secured to the right frontal dura matter (Figure 1). Intravenous fentanyl infusion (10 µg/kg/h) was then started about 10 min prior to baseline data acquisition to provide analgesia for the duration of the study.

### 2.3. Data Acquisition

Optical neuromonitoring of the left frontal cortex was performed with interleaved FD-DOS and bDOS data acquisition (Section 2.3.1). Baseline measurements were obtained immediately prior to the initiation of the 4 h IV infusion period (Figure 1). During baseline, 5 min of bDOS data was acquired first, followed by 5 min of FD-DOS data. During the IV infusion period (Figure 1), the monitoring comprised 28 min of bDOS acquisition interleaved with 2 min of FD-DOS acquisition. Continuous laser Doppler monitoring of baseline-normalized relative cerebral blood flow (i.e., rCBF = CBF/CBF_Baseline_) was performed in parallel. Cerebral microdialysis sampling (Section 2.3.2) was acquired at the end of the baseline period and every 30 min during the IV infusion period. Finally, systemic arterial and venous blood gas samples were drawn at the end of baseline, and at 30 min, 60 min, 120 min, 180 min, and 240 min during the IV infusion period. Per blood gas, we examined the venous lactate concentration, arterial blood oxygen saturation (SaO_2_), partial arterial oxygen pressure (PaO_2_), and hematocrit (Hct). Arterial blood oxygen content (CaO_2_) was readily calculated from the latter metrics (see Supplementary Material) [19].

#### 2.3.1. Optical Neuromonitoring

FD-DOS and bDOS measurements were acquired with a commercially available system (MetaOx, ISS Inc, Champaign, IL, USA) and a custom-built system, respectively, using a single optical probe secured to the forehead (Figure 1, the probe is described in detail in Supplementary Material). The FD-DOS system comprised 16 intensity modulated lasers (4 lasers at each of 4 wavelengths, i.e., 680, 760, 805, 830 nm; modulation frequency, 110 MHz modulation) and 4 PMT detectors. (Note, the MetaOx is also capable of diffuse correlation spectroscopy (DCS) monitoring of blood flow; regrettably, DCS data quality was not sufficient for analysis due to low signal intensity (<5000 detected photons a second)). The bDOS system comprised a fiber-coupled halogen lamp (HL-2000-HP-FHSA, Ocean Optics, Dunedin, FL, USA) and a fiber-coupled custom f/1.5 high throughput spectrometer (650–1050 nm spectral range, 100 µm slit width, 4.8 nm FWHM resolution, TEC cooled to −15 °C; Wasatch Photonics, Morrisville, NC, USA).

FD-DOS and bDOS acquisition were sequentially interleaved as shown in Figure 1. During FD-DOS acquisition, the bDOS lamp shutter was closed, and during bDOS acquisition, the FD-DOS lasers and detectors were shut off. The FD-DOS and bDOS measurements were also calibrated before and after each monitoring session using a solid phantom with known optical properties and a spectrally flat reflectance standard (see Supplementary Material) [32,33,34].

Cerebral measurements of OEF, tissue blood oxygen saturation (StO_2_), total hemoglobin concentration (HbT), and differential change in the concentration of oxCCO relative to baseline (ΔoxCCO) were computed once every minute (see Supplementary Material). Note, optical monitoring specifically detects changes in the CuA center redox state in CCO; we do not probe CuB or other metallic centers. Note also, the OEF estimate accounts for CaO_2_ changes. All concentration measurements are per volume of tissue.

#### 2.3.2. Cerebral Microdialysis

The cerebral microdialysis catheter is a selectively permeable membrane which permits the diffusion of small molecule biomarkers from interstitial fluid in the brain into circulating saline perfusate [30]. Normal saline was infused through the catheter (CMA 71 Elite, mDialysis AB, Stockholm, Sweden) at a rate of 1 µL/min, and the resulting dialysate was collected in a microvial. Microvial samples were stored and replaced every 30 min to examine temporal changes in collected biomarkers (Figure 1). The samples were analyzed to determine their concentrations of lactate, pyruvate, and glycerol using an automated ISCUS Flex^TM^ Microdialysis Analyzer (mDialysis AB, Stockholm, Sweden). We also computed the lactate–pyruvate ratio (LPR) in each sample. Elevated LPR and elevated glycerol are often used as indications of increased anaerobic metabolism and cell injury, respectively [30].

### 2.4. Statistical Analysis

The statistical analyses were performed in MATLAB R2022a (Mathworks Inc., Natick, MA, USA) with *p* ≤ 0.05 indicating significance. First, to assess differences between baseline physiology across experimental groups, we performed Kruskal–Wallis tests; if significance was found, we used a post hoc Wilcoxon rank-sum test between each group.

Our primary analyses investigated the temporal changes in oxCCO, OEF, and LPR during the 4 h IV infusion period in each experimental group. We first computed the time-averages of the differential changes from baseline in oxCCO and OEF across the 5 min intervals before the microdialysis sampling points, i.e., denoted as ΔoxCCO and ΔOEF; this scheme provided uniform sampling of the optical and microdialysis metrics. Then, during the IV infusion period, we performed both simple linear and 2-period piecewise linear mixed effects regressions of ΔoxCCO vs. time, ΔOEF vs. time, and LPR vs. time for the rotenone + placebo and rotenone + prodrug groups. The time periods of the piecewise linear regressions were 0–2 h and 2–4 h. For the ΔoxCCO and ΔOEF regressions, random slopes were incorporated for each animal to account for individual variations in the trends (the intercept was forced to zero in the regressions because ΔoxCCO and ΔOEF are, by definition, zero at time zero). For the LPR regressions, random slopes and intercepts were incorporated for each animal.

Our rationale for using the 2-period piecewise regression is that it is among the simplest of analyses to account for a possible time-dependent effect. Such time-dependence could arise because the total delivered dose of rotenone and succinate prodrug increases with infusion time, and because higher doses (longer infusion times) may alter physiological responses. In the rotenone + placebo group, the adjusted R^2^ values were larger for the simple linear regressions than for the piecewise linear regressions. The reverse was true for the rotenone + prodrug group. Thus, we present simple linear regressions for the rotenone + placebo group, and piecewise linear regressions for the rotenone + prodrug group. Note, for the control group, we only performed simple linear mixed effects regressions.

In secondary analyses, we also evaluated the temporal changes in ΔHbT, StO2, mean arterial blood pressure (MAP), venous lactate, cerebral blood flow (measured with laser Doppler), Hct, CaO_2_, and additional microdialysis metrics (i.e., lactate, pyruvate, glycerol). As in the primary analyses, simple linear mixed effects regressions were performed for the control and rotenone + placebo groups, and 2-period piecewise linear mixed effects regressions were performed for the rotenone + prodrug group. Finally, for each experimental group, we used simple linear mixed effects regression analyses to estimate the associations between LPR and ΔoxCCO, LPR and ΔOEF, and ΔOEF and ΔoxCCO.

## 3. Results

No animals were excluded. Pre-infusion baseline summary statistics for the cerebral optical metrics, cerebral microdialysis metrics, and systemic lactate were similar between groups (Table 1). There was a marginally significant difference in baseline MAP between groups (*p* = 0.05). From post hoc tests, MAP was found to be lower in the rotenone + placebo group compared to the control group (*p* = 0.02). MAP was not different between the rotenone + placebo and rotenone + prodrug groups, however, and thus MAP should not be a confounder per the differing temporal trends in optical and microdialysis metrics between those groups.

**Figure 2:**
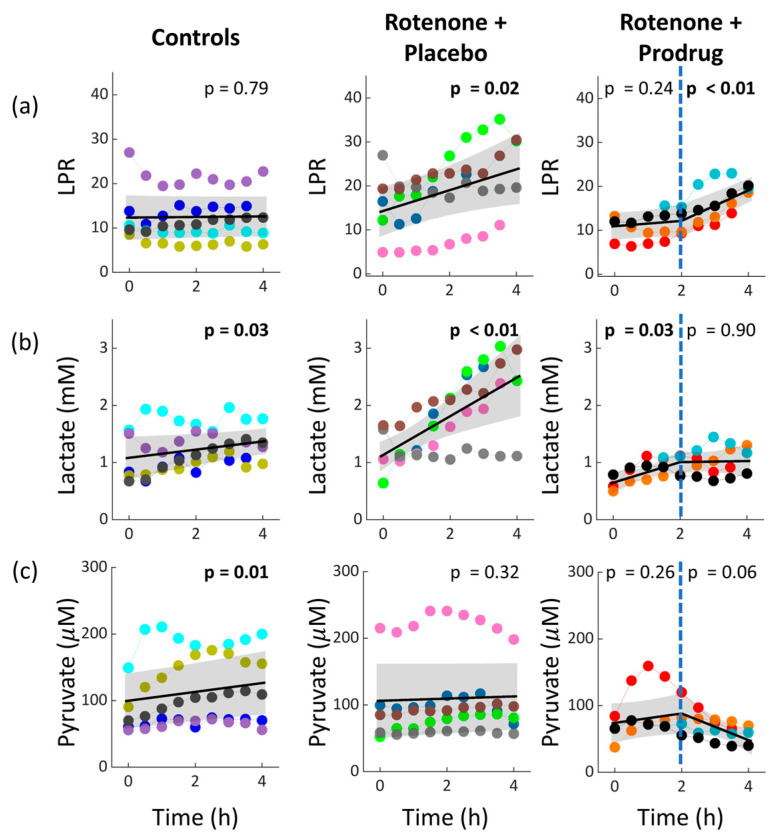
Temporal trends of cerebral microdialysis LPR (**a**), lactate (**b**), and pyruvate (**c**) metrics during the 4 h infusion period of each experimental group (infusion begins at time zero). Each color represents a different subject. In the rotenone + prodrug group, LPR data for one of the five subjects is missing due to instrument malfunction (there are also three missing datapoints in another subject, light blue color). In all plots, the linear regression fit (solid line) with its 95% CI (shaded region) is shown, along with the *p*-value for the null hypothesis of zero slope. The fit is simple linear for the control and rotenone + placebo groups, and 2-period piecewise linear (periods separated by dashed vertical line) for the rotenone + prodrug group (see Section 2.4).

The cerebral microdialysis measurements of LPR, lactate, and pyruvate as a function of time for each group are shown in Figure 2 (the glycerol metric is not plotted, but its regression slopes are tabulated in Table 2). As expected, LPR remained constant in the control group and increased in the rotenone + placebo group (*p* = 0.02). The slope of the rotenone + placebo group increase, 2.3 per hour, shows a clinically meaningful change, i.e., LPR approaches abnormal levels (>20) within a few hours [35]. LPR also increased, however, during the last two hours of infusion in the rotenone + prodrug group (*p* < 0.01) to levels of about 20.

Interestingly, the LPR increase was driven by increasing lactate in the rotenone + placebo group, but in the rotenone + prodrug group, the LPR increase was driven by decreasing pyruvate (in combination with stabilizing lactate levels). The lactate increase in the rotenone + placebo group suggests increased anaerobic metabolism caused by rotenone-induced mitochondrial dysfunction. The pyruvate decrease and stabilized lactate in the rotenone + prodrug group suggest increased mitochondrial oxygen metabolism during the last two hours of infusion (see Section 4).

Small but significant cerebral lactate increases were observed in the control group (*p* = 0.03) and during the first two hours of infusion in the rotenone + prodrug group (*p* = 0.03). The control group increase might be a consequence of decreasing MAP (see Table 2), while the rotenone + prodrug group increase might reflect the residual lactate production following exposure to rotenone and prodrug. Glycerol concentration was approximately constant in all groups (Table 2), which suggests that cell membrane damage did not occur.

**Figure 3:**
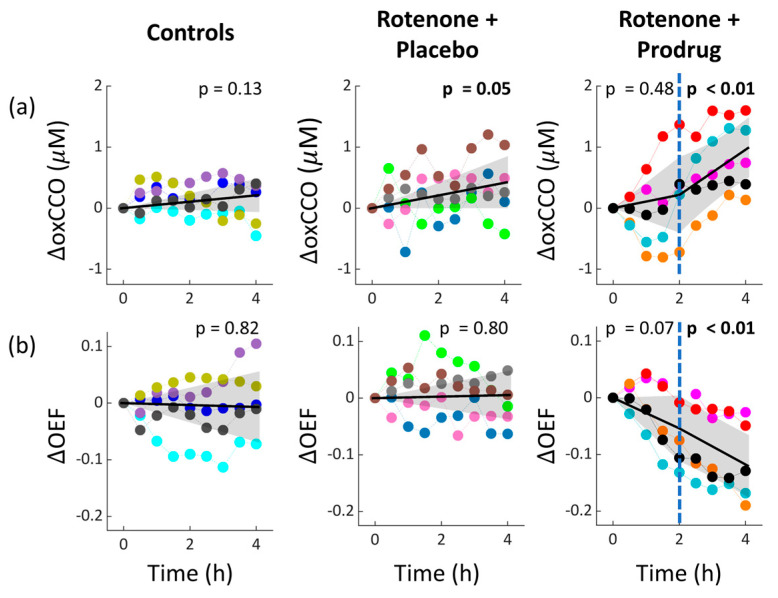
Temporal trends of cerebral ΔoxCCO (**a**) and ΔOEF (**b**) during the 4 h infusion period of each experimental group (infusion begins at time zero). Each color represents a different subject, and the points are five-minute averages of data prior to microdialysis sampling times (see Figure 1). In all plots, the linear regression fit (solid line) with its 95% CI (shaded region) is shown, along with the *p*-value for the null hypothesis of zero slope. The fit is simple linear for the control and rotenone + placebo groups, and 2-period piecewise linear for the rotenone + prodrug group (see Section 2.4).

The concurrent optical cerebral ΔoxCCO and ΔOEF data are plotted in Figure 3. oxCCO and OEF were constant in the control group. In the rotenone + placebo group, oxCCO marginally increased (*p* = 0.05), but OEF unexpectedly remained constant (*p* = 0.80). Lastly, the rotenone + prodrug group exhibited a pronounced increase in oxCCO (*p* < 0.01) and decrease in OEF (*p* < 0.01).

**Figure 4:**
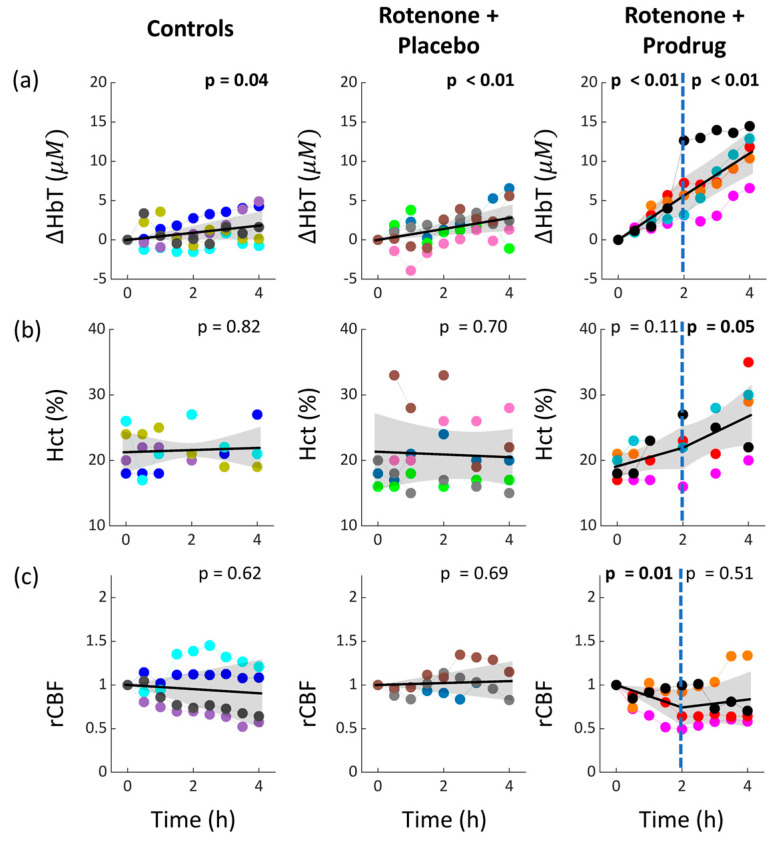
Temporal trends of cerebral ΔHbT (**a**), Hct (**b**), and rCBF (**c**) during the 4 h infusion period of each experimental group (infusion begins at time zero). Note, laser Doppler measures the baseline-normalized rCBF. Each color represents a different subject, and the ΔHbT and rCBF points are five-minute averages of data prior to microdialysis sampling times. In all plots, the linear regression fit (solid line) with its 95% CI (shaded region) is shown, along with the *p*-value for the null hypothesis of zero slope. The fit is simple linear for the control and rotenone + placebo groups, and 2-period piecewise linear for the rotenone + prodrug group. rCBF data was available for only four, three, and four of the five subjects, respectively, in the control, rotenone + placebo, and rotenone + prodrug groups.

In addition, hematocrit (Hct) substantially increased in the rotenone + prodrug group (Figure 4), which resulted in pronounced increases in optically measured total hemoglobin concentration (HbT). In the other two groups, small HbT increases were observed that may reflect a vasodilation response to decreasing MAP (Table 2). Laser Doppler showed constant rCBF in the control and placebo groups, and decreasing rCBF during the first two hours of infusion in the prodrug group.

Finally, the direct associations (i.e., linear regressions slopes) between the metabolism metrics for each experimental group are reported in Table 3. In the rotenone + prodrug group, LPR increases were linearly associated with an increase in oxCCO (*p* < 0.01) and a decrease in OEF (*p* < 0.01). In the other two groups, LPR increases were not associated with changes in the optical metrics. Similarly, oxCCO increases were linearly associated with OEF decreases only in the rotenone + prodrug group (*p* < 0.01).

## 4. Discussion

We performed non-invasive optical neuromonitoring and invasive microdialysis neuromonitoring in swine to assess metabolic changes induced by rotenone inhibition of mitochondrial complex I and NV354 succinate prodrug treatment. Our use of a swine model with clinically relevant molecular and physiologic responses, and our technical innovations to monitor the metabolic responses (discussed below), are strengths of this work. Our pilot findings motivate future research into using succinate supplementation to treat complex I dysfunction. The optical neuromonitoring used herein also shows promise to provide an additional outcome metric for adaptive trial design of patients with mitochondrial disease, but future improvements in the technology’s brain sensitivity are needed to improve its ability to monitor small metabolic changes.

### 4.1. Cerebral Physiological Response to Rotenone Exposure

Microdialysis evidence, i.e., increasing LPR driven by increasing lactate concentration, supports the expectation that rotenone exposure impairs mitochondrial complex I. The cerebral lactate increase is a consequence of the increase in anaerobic metabolism that is needed to compensate for decreased oxygen metabolism. The optically measured oxCCO increase, however, was only marginally significant (*p* = 0.05), and the oxCCO data were not directly associated with the LPR changes. In addition, we had expected that diminished aerobic metabolism would result in less oxygen extraction from the vasculature (or decreased OEF), but the OEF optical metric remained constant.

The lack of robust optical detection of the complex I impairment, e.g., compared to detection by microdialysis, could be a consequence of extra-cerebral tissue contamination (e.g., from scalp and skull) and experimental noise, both of which reduce the sensitivity of optics to cerebral metabolic changes [36,37]. Specifically, we expect that extra-cerebral tissues have a much lower metabolic rate of oxygen than the brain, and thus extra-cerebral tissues may not be impacted significantly by rotenone. Accordingly, extra-cerebral tissue contamination can lead to underestimation of the cerebral metabolic effect, which makes it more challenging to separate modest metabolic effects from experimental noise (the metabolic impairment from rotenone was not sufficiently severe to induce acute elevations in cerebral interstitial glycerol concentration). Prior work has suggested that oxCCO, which directly probes mitochondria, is less confounded by extra-cerebral tissue contamination because of much higher concentrations of mitochondria in the brain compared to the skull or scalp [36,38,39]. Thus, our observations of increased oxCCO and constant OEF could be a consequence of the improved brain sensitivity of the oxCCO metric.

**Figure 5:**
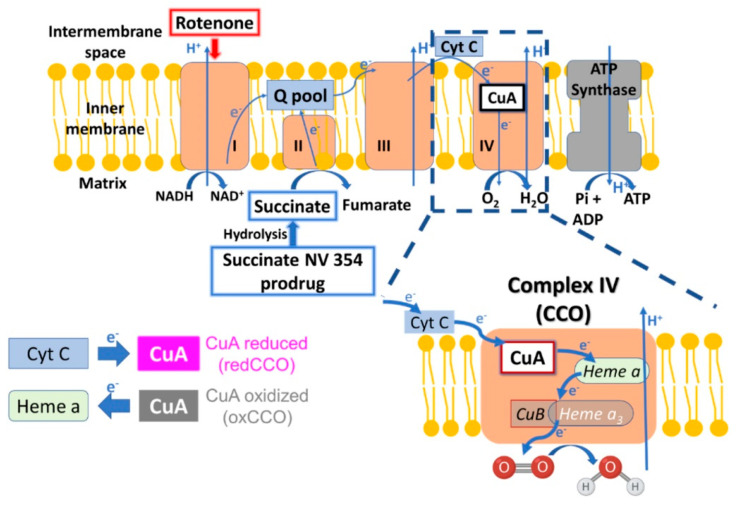
Schematic of the mitochondrial electron transport chain. In normal mitochondrial function, electrons (e^−^) from complex I and complex II ultimately flow through cytochrome-c-oxidase (CCO; or complex IV) to reduce O_2_ to H_2_O, and pump protons (H^+^) into the intermembrane space. ATP Synthase uses the resultant H^+^ gradient to generate ATP. CCO is reduced and oxidized by electron in-flow and out-flow, respectively (more precisely, it is the reduced and oxidized form of CuA, the di-copper center cofactor in CCO, which is monitored using optics). Change in the electron in-flow rate relative to the out-flow rate alters the redox ratio of CuA from its equilibrium state. Rotenone impairs function by inhibiting complex I (the electron out-flow rate from complex I is decreased). Succinate NV354 prodrug offers a possible means to restore function by increasing the electron out-flow rate from complex II. Specifically, after passive entry into cells from the vasculature, NV354 is hydrolyzed to release succinate, which increases succinate concentration in the mitochondrial matrix. Other electron carriers shown include the coenzyme Q and cytochrome-c protein.

In addition, the change in oxCCO concentration depends on the difference between the electron in-flow rate to CCO and electron out-flow rate from CCO (see Figure 5). Since complex I inhibition lowers mitochondrial metabolism, one might anticipate a decrease in both the electron in-flow rate to CCO and the out-flow rate from CCO. Thus, even if the absolute reduction in the in-flow rate is substantial, the net difference between the in-flow and out-flow rates could be smaller. This is another way for rotenone-induced complex I inhibition to have a smaller effect on oxCCO concentration compared to its effect on cerebral lactate. As to why oxCCO increased, the out-flow rate during complex I inhibition might be sustained by normal intracellular oxygen concentrations. Indeed, in patients with acute brain injury, there is evidence of an association between oxygen concentration and the CCO redox state [40,41]. Thus, although isolated rat liver and pigeon heart mitochondria experiments show that non-hypoxic intracellular oxygen concentrations (i.e., above ~10 mmHg [42,43] and ~1 mmHg [44] thresholds) do not influence the redox state of CCO in healthy mitochondria, the rotenone-induced impairment could yield “unhealthy” mitochondria that have altered relationships between CCO redox state and oxygen.

### 4.2. Cerebral Physiological Response to Succinate Prodrug Treatment

Although we did not directly monitor mitochondrial oxygen metabolism (see Section 4.4), our microdialysis metrics suggest that NV354 prodrug treatment increased oxygen metabolism during rotenone infusion. Specifically, cerebral interstitial lactate levels stabilized later in the infusion period; this observation suggests that cerebral lactate production was normalized because of increased oxygen metabolism and increased generation of ATP from mitochondria (note, the stabilization of lactate also coincided with pyruvate decreases; since pyruvate is oxidized to acetyl-CoA during aerobic metabolism, an increase in aerobic metabolism could decrease pyruvate levels). In addition, the increase in cerebral lactate during the first 2 h of prodrug infusion appears to be attenuated compared to the increase in cerebral lactate from rotenone infusion alone (unfortunately, we are not powered to show a significant difference). The complete stabilization of cerebral lactate during the second 2 h of infusion might be due to the accumulation of larger intracellular succinate levels. Specifically, since the total drug dose delivered increases with infusion time, the drug might only reach therapeutic levels inside cells during the second 2 h of treatment.

Our results also illustrate the complexities of interpreting the microdialysis LPR metabolism metric, which was observed to increase in both the rotenone + placebo and rotenone + prodrug groups. Specifically, an increase in LPR driven by increased lactate reflects glycolytic upregulation and impaired oxygen metabolism. An increase in LPR driven by decreased pyruvate and stabilized lactate levels, by contrast, suggests enhanced oxygen metabolism. Thus, changes in LPR are best interpreted in the context of the separate, independent changes in lactate and pyruvate concentration.

The observed OEF decrease in the rotenone + prodrug group is likely explained by the increased arterial blood oxygen content (CaO_2_) that arose from increased hematocrit, i.e., OEF will decrease if the increase in cerebral oxygen delivery exceeds the increase in cerebral oxygen metabolism [19]. The observed hematocrit increase was puzzling and unexpected, and a future study is needed to confirm and understand the effect. Note, the hematocrit increase should not affect the accuracy of our optical metrics (see Supplementary Material), but as we just noted, it is an important factor for interpreting OEF. Note also, given the short temporal monitoring period and the constant hematocrit in the placebo group, it is unlikely that the number of red blood cells is increasing during prodrug administration. Instead, the increased hematocrit reflects a decrease in plasma volume (from increased fluid excretion). The rCBF decrease observed with laser Doppler during the first two hours of infusion was also unexpected and might be a consequence of increased CaO_2_. However, the laser Doppler blood flow measurements may also have artifacts from vasculature in the dura matter [45] since the laser Doppler probe was resting on top of the dura.

Finally, we expect that increased oxygen metabolism is accompanied by increases in both the electron in-flow rate to CCO and the electron outflow rate from CCO. The oxCCO increase we observed indicates that the out-flow rate increased more than the in-flow rate (see Figure 5). This could be explained by a prodrug-induced decrease in the proton electrochemical potential across the inner mitochondrial membrane and a resultant increase in ATP synthesis; prior work has suggested that this effect will disproportionately increase the electron out-flow rate [46]. Future work is needed to better understand and test predictions about the relation between oxCCO concentration and metabolic demand in vivo, given the different factors at play in our study.

### 4.3. Importance of the Analysis Algorithm for oxCCO Measurements

Our optical analysis algorithm leveraged photon diffusion theory to quantify changes in oxCCO (see Supplementary Material). In contrast to the modified Beer-Lambert schemes that are commonly used for these measurements [13,14,15,17,47,48,49], photon diffusion theory more readily accounts for changes in tissue scattering [16,18,50,51,52]. In our experiments, we did indeed observe significant tissue scattering changes over time. Specifically, in the rotenone + placebo group, the Mie scattering parameters, A (*p* < 0.01) and b (*p* = 0.02), both decreased over time, and in the rotenone + prodrug group, b decreased over time (*p* = 0.02). Importantly, we found that if the modified Beer-Lambert scheme was used for analysis, then the estimated ΔoxCCO temporal change was no longer significant in the rotenone + placebo group (i.e., *p* = 0.80). Further, although the modified Beer-Lambert scheme still showed 2nd period increases in ΔoxCCO for the rotenone + prodrug group, the magnitude of the increase was smaller than that of the photon diffusion theory scheme (i.e., a slope of 0.08 µM/h (*p* = 0.04) versus 0.19 µM/h (*p* < 0.01) obtained with photon diffusion theory). These findings thus suggest the importance of employing full photon diffusion theory for oxCCO measurement if tissue scattering changes.

### 4.4. Limitations

The sample size of our study is small (n = 5 in each group), and therefore our findings need confirmation from a larger study. Second, we lack direct evidence of increased pyruvate usage in the tricarboxylic acid (TCA) cycle and increased oxidative ATP production during prodrug treatment. In future work, such evidence could be gleaned from cerebral metabolomics analysis of the metabolites involved in glycolysis and TCA cycles [53].

Third, the co-treatment paradigm at a single prodrug dose regiment limits direct therapeutic interpretation. However, the stabilization of cerebral lactate levels observed with microdialysis suggests that NV354 prodrug can maintain oxidative capacity under metabolic stress. Although reversing acute rotenone effects in healthy swine is not equivalent to treating long-standing disease, this proof-of-concept is essential before testing rescue therapy protocols. It also motivates future dose–response investigations in swine.

Fourth, the present study does not have an NV354-only experimental group. We note that in a prior study of rodents [28], NV354 treatment alone did not show any differences (compared to shams) in MAP, venous blood gases, and cerebral mitochondrial respirometry. Thus, we expect that our findings of baseline stability in our control group will be similar to those that result from the administration of NV354 treatment alone. That said, a future study of the effects of NV354 treatment alone in swine needs to be conducted.

Fifth, our study does not relate the acute effects of prodrug treatment to longer-term therapeutic effects. Showing this relation will be critical for translation, but our study design is still relevant. Acute intervention efficacy is a prerequisite for longer-term benefits.

Finally, to derive the optical metrics, we have assumed homogeneous tissue optical properties and constant total CCO concentration. The former assumption can lead to error from extra-cerebral tissue contamination (see Section 4.1), while the latter can lead to error in the oxCCO metric if the tissue mitochondria density changes. The magnitude of the errors arising from these effects, as well as possible errors from crosstalk between oxCCO and hemoglobin during spectroscopic inversion, should be explored in future work. Note, it is possible that the latter crosstalk could contribute to the oxCCO increases observed.

## 5. Conclusions

We used diffuse optical and cerebral microdialysis techniques to assess the metabolic effects of a novel succinate prodrug (NV354) in swine with rotenone-induced mitochondrial complex I dysfunction. Cerebral microdialysis is a well-established technique for monitoring metabolism via its invasive measurements of lactate, pyruvate, and lactate–pyruvate ratio (LPR) in the interstitial fluid of the brain parenchyma. The diffuse optical techniques, i.e., frequency-domain and broadband diffuse optical spectroscopy, provide non-invasive and continuous access to cerebral oxygen extraction fraction (OEF) and oxidized cytochrome-c-oxidase concentration changes (ΔoxCCO).

Our results support the expectation that rotenone exposure induces cerebral mitochondrial dysfunction in swine. Specifically, rotenone exposure increased LPR, lactate and oxCCO concentrations. Our results also suggest that administering NV354 during rotenone exposure can maintain mitochondrial function under stress. Although proof of increased oxygen metabolism induced by NV354 treatment is lacking, the observed stabilization of cerebral lactate is consistent with the normalization of cerebral lactate production due to increased oxygen metabolism.

Finally, to our knowledge, this study provides the first in vivo optical measurements of oxCCO and OEF changes induced by primary mitochondrial dysfunction and mitochondria-targeted drugs. Notably, our findings suggest that the oxCCO metric of metabolism, which directly probes the mitochondria, may be more sensitive to mitochondrial dysfunction than the OEF metric that probes tissue vasculature. Interpreting the oxCCO metric, however, is challenging. Our work suggests that it increased in both the placebo group and the prodrug group for different reasons, which illustrates the value of multimodal neuromonitoring.

## Figures and Tables

**Table 1:** Pre-infusion baseline measurements presented as medians and interquartile ranges (*p* values from Kruskal–Wallis tests). The tissue scattering amplitude (A) and power (b) are defined in the Supplementary Material.

Parameter	Controls	Rotenone + Placebo	Rotenone + Prodrug	*p*
Cerebral Optics				
OEF	0.63 (0.59, 0.66)	0.58 (0.54, 0.60)	0.58 (0.56, 0.63)	0.53
HbT (µM)	49.0 (44.4, 58.3)	49.8 (49.1, 55.2)	47.1 (46.6, 51.6)	0.57
StO_2_	0.52 (0.49, 0.54)	0.55 (0.53, 0.58)	0.56 (0.51, 0.57)	0.47
A (cm^−1^)	14.6 (14.2, 16.4)	15.4 (13.7, 16.3)	14.3 (13.7, 15.9)	0.89
b	1.0 (0.93, 1.1)	0.96 (0.89, 1.0)	1.0 (0.95, 1.1)	0.59
Cerebral Microdialysis				
Lactate (mM)	0.84 (0.75, 1.5)	1.6 (0.95, 1.6)	0.59 (0.52, 0.74)	0.08
Pyruvate (µM)	70.2 (59.5, 105)	85.1 (57.0, 128)	65.6 (44.7, 79.6)	0.59
LPR	10.5 (9.34, 17.1)	16.5 (10.4, 21.3)	12.0 (8.23, 13.0)	0.60
Glycerol (µM)	17.4 (14.8, 20.9)	22.5 (18.3, 26.7)	9.83 (8.89, 14.2)	0.08
Systemic				
Venous Lactate (mM)	1.1 (0.79, 1.3)	1.0 (0.75, 1.3)	1.0 (0.79, 1.7)	0.91
MAP (mmHg)	73.6 (62.1, 83.3)	56.3 (52.7, 58.0)	66.8 (56.5, 78.7)	0.05
Hct (%)	22.0 (19.0, 25.0)	18.5 (17.5, 20.0)	18.0 (17.0, 20.2)	0.25
SaO_2_ (%)	97.0 (96.5, 98.0)	97.0 (96.8, 97.2)	98.0 (97.8, 98.2)	0.14
PaO_2_ (mmHg)	90.0 (80.5, 102.0)	91.0 (82.5, 99.5)	95.0 (91.8, 111.8)	0.31
CaO_2_ (mL O_2_/dL blood)	9.44 (8.25, 10.8)	8.03 (7.62, 8.62)	7.98 (7.46, 8.86)	0.28

**Table 2:** Slopes (in per hour units) of the linear mixed effects regression fits of MAP, microdialysis glycerol, systemic venous lactate, and CaO_2_ versus infusion time for each experimental group (see Table 1 for metric units). Note, there are two slopes in the piecewise fit for the prodrug group (period 1, 0–2 h of infusion; period 2, 2–4 h of infusion). The *p*-values for the null hypothesis of zero slope are also included.

	Controls		Rotenone + Placebo		Rotenone + Prodrug (Period 1|Period 2)			
	Slope (95% CI)	*p*	Slope (95% CI)	*p*	Slope (95% CI)	*p*	Slope (95% CI)	*p*
MAP	−3.9 (−6.4, −1.5)	<0.01	−2.3 (−3.2, −1.3)	<0.01	−3.0 (−8.6, 2.5)	0.3	2.2 (−2.9, 7.3)	0.4
Glycerol	0.21 (−0.85, 1.3)	0.7	0.37 (−0.81, 1.6)	0.5	−2.2 (−7.6, 3.2)	0.4	0.85 (−1.4, 3.1)	0.4
Venous Lactate	−0.03 (−0.17, 0.11)	0.6	0.25 (−0.01, 0.51)	0.06	0.32 (0.10, 0.52)	<0.01	0.94 (0.12, 1.76)	0.03
CaO_2_	0.10 (−0.53, 0.73)	0.7	0.12 (−0.39, 0.63)	0.6	0.59 (−0.18, 1.37)	0.1	0.94 (−0.12, 2.00)	0.08

**Table 3:** Linear associations between changes in metabolism metrics during the 4 h infusion period of each experimental group. The linear mixed effects regression slope (95% confidence interval) and the *p*-value for the null hypothesis of zero slope are reported. Note, the slope units for the two ΔoxCCO associations are 1/µM, and the slope unit for LPR vs. ΔOEF is dimensionless.

	Controls		Rotenone + Placebo		Rotenone + Prodrug	
Association	Slope (95% CI)	*p*	Slope (95% CI)	*p*	Slope (95% CI)	*p*
LPR vs. ΔoxCCO	−0.96 (−5.6, 3.7)	0.68	0.67 (−6.6, 7.9)	0.93	4.9 (3.2, 6.7)	<0.01
LPR vs. ΔOEF	−5.4 (−22, 11)	0.51	−0.76 (−59, 58)	0.98	−54 (−74, −35)	<0.01
OEF vs. ΔoxCCO	0.04 (−0.01, 0.09)	0.12	−0.0005 (−0.026, 0.025)	0.97	−0.06 (−0.10, 0.02)	<0.01

## Data Availability

The data and code presented in this study are available on request from the corresponding author. The data are not publicly available due to privacy.

## Supplementary Materials

The following supporting information can be downloaded at: https://www.mdpi.com/article/10.3390/metabo16010065/s1, Figure S1: Schematic of the 8-step algorithm used to derive absolute cerebral tissue absorption spectra from hybrid broadband diffuse optical spectroscopy (bDOS) and frequency-domain diffuse optical spectroscopy (FD-DOS) data; Figure S2: Example one-minute average of broadband spectral measurements acquired on a pig during the study’s baseline period; Figure S3: Schematic of the two potential routes of hydrolysis of the NV354 succinate prodrug once it is inside the cell; Figure S4: Metabolite ratios detected by magnetic resonance spectroscopy in the basal ganglia after 6 h of rotenone infusion. Reference [54] is cited in the supplementary materials.
