# Supplementary material for: Optical and Microdialysis Monitoring of Succinate Prodrug Treatment in a Rotenone-Induced Model of Mitochondrial Dysfunction in Swine
Source: Metabolites. 2026 Jan 11;16(1):65. doi: 10.3390/metabo16010065 (PMC12844242; doi:10.3390/metabo16010065)
Supplement: Supplementary file 1 [file metabolites-16-00065-s001.zip › metabolites-4079329-supplementary.pdf]

Optical and Microdialysis Monitoring of Succinate Prodrug Treatment in a Rotenone-Induced Model of Mitochondrial Dysfunction in Swine

Alistair Lewis, Rodrigo M. Forti, Tiffany S. Ko, Eskil Elmér, Meagan J. McManus, Arjun G. Yodh, Todd J. Kilbaugh, Wesley B. Baker

Supplementary Material

S.1 Optical Probe and Data Acquisition

A schematic of the optical probe is shown in Figure 1 (main text). The probe includes two FD-DOS source positions, two FD-DOS detector positions, one bDOS source position, and one bDOS detector position. The FD-DOS source-detector separations were 1.5, 2.0, 2.5, and 3.0 cm, and the bDOS source-detector separation was 3.0 cm. Light was delivered/collected at each position with optical fiber bundles. The bDOS halogen lamp and spectrometer were coupled to 2.5-mm diameter and 1.0-mm diameter 90°-bend borosilicate bundles, respectively (0.66 NA, Fiberoptics Technology Inc, Pomfret, CT, USA). 90°-bend borosilicate bundles (2.5 mm diameter, 0.66 NA) were also used to connect each FD-DOS source position to 4 intensity modulated lasers in the MetaOx, *i.e.,* via coupling to 4×1 fan-out fiber bundles inside the enclosure (one laser at each wavelength). Finally, FD-DOS detection was performed through 3-mm right-angle prisms glued to borosilicate fiber bundles (2 mm diameter, 0.55 NA, Fiberoptic Systems, Simi Valley, CA, USA).

During FD-DOS acquisition, time-division source multiplexing was used; 0.02 s was required to cycle all wavelengths through the two source positions. For each light wavelength and source position, the amplitude and phase of the measured diffuse photon density waves at the detectors were recorded. For bDOS acquisition (spectrometer integration time, 100 ms), an optical shutter in the lamp was used to interleave 1 s of tissue diffuse reflectance measurements (shutter open) with 1 s of dark count measurements (shutter closed).

Before and after each monitoring session, the FD-DOS measurements were calibrated using a solid phantom with known optical properties to estimate the light coupling coefficients to tissue for each fiber bundle (ISS Inc, Champaign, IL, USA).[1] Furthermore, the spectral response function of the bDOS system was determined by using a custom 3-D printed holder to position the probe on a spectrally flat reflectance standard (SRS-40-020, Labsphere, North Sutton, NH, USA).

S.2 Optical Analysis Algorithm: Determination of Broadband Absorption Spectra

Absolute cerebral tissue absorption spectra were derived from the FD-DOS and bDOS measurements in eight steps (Figure S1). This derivation of the absorption spectra follows an approach similar to one described in prior work;[2,3] we implemented the approach in MATLAB R2022a (Mathworks Inc., Natick, MA, USA).

In step 1, the bDOS reflectance, $R(\lambda_{BB}, t)$, is derived from temporal one-minute averages (no overlap) of the raw data corrected for dark counts and for the instrument's spectral response function (t denotes the midpoint time of each 1-minute time-average). In Figure S1, we denote the bDOS measured dark count and raw tissue diffuse reflectance spectra at each measurement timepoint, t_m, as $R_{dark}(\lambda_{BB}, t_m)$ and $R_{raw}(\lambda_{BB}, t_m)$; the broadband wavelengths (λ_{BB}) span from 650 to 1000 nm. We also define $R_{RefStd}(\lambda_{BB})$ as the spectral response function obtained from reflectance standard measurements. Then, $R(\lambda_{BB}, t) = \langle R_{raw}(\lambda_{BB}, t_m) - R_{dark}(\lambda_{BB}, t_m)\rangle_t / R_{RefStd}(\lambda_{BB})$. Here, the $\langle\,\rangle_t$ brackets denote a 1-minute time-average centered at time *t*.

In step 2, interleaved FD-DOS measurements of the cerebral tissue optical absorption and reduced scattering coefficients, *i.e.,* $\mu_a(\lambda_{FD}, t_{FD})$ and $\mu_s'(\lambda_{FD}, t_{FD})$, respectively, were obtained at time t_{FD} and wavelength λ_{FD}. Each measurement of tissue optical properties entailed fitting the phantom-calibrated FD-DOS measurements of amplitude and phase (2 Hz sampling) to the frequency-domain

photon diffusion equation solution for a semi-infinite homogeneous medium.[4] These absorption and scattering properties, obtained at 2 Hz, were further averaged over the ~2-minute FD-DOS acquisition interval centered at time t_{FD}. Frequency-domain values for the tissue scattering amplitude and power, *i.e.*, $A(t_{FD})$ and $b(t_{FD})$, were derived by fitting the measured $\mu_s'(\lambda_{FD}, t_{FD})$ to a wavelength-dependent power law model for tissue scattering (the power law model is empirical but has been related to Mie scattering theory models):[4] $\mu_s'(\lambda_{FD}, t_{FD}) = A(t_{FD})(\lambda_{FD}/(500\text{ nm}))^{-b(t_{FD})}$.

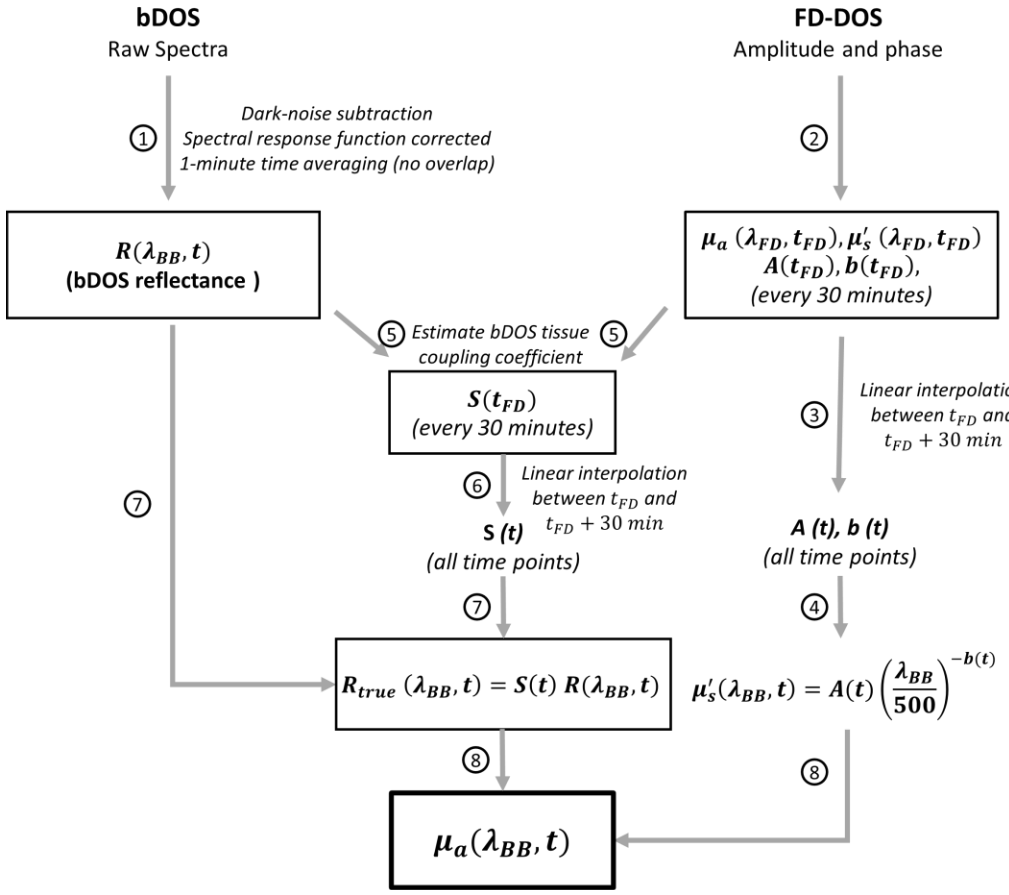

Figure S1. Schematic of the 8-step algorithm used to derive absolute cerebral tissue absorption spectra from hybrid broadband diffuse optical spectroscopy (bDOS) and frequency-domain diffuse optical spectroscopy (FD-DOS) data. μ_a and μ_s' are the wavelength-dependent absorption and reduced scattering coefficients. λ_{FD} denotes the FD-DOS source wavelengths (680, 760, 805 and 830 nm), λ_{BB} denotes the bDOS wavelengths (650-1000 nm); t_{FD} denotes the midpoint times of the 2-minute FD-DOS acquisition intervals (see Figure 1, main text), t denotes the midpoint times of the 1-minute averaged bDOS spectra; A is the Mie scattering amplitude and b is the Mie scattering power, which are assumed to be independent of wavelength; S is the multiplicative factor that converts the bDOS reflectance ($R(\lambda_{BB}, t)$) to the "true" tissue reflectance ($R_{true}(\lambda_{BB}, t)$) predicted by photon diffusion theory. For each λ_{BB}, $R_{true}(\lambda_{BB}, t)$ was fit to the continuous-wave semi-infinite photon diffusion Green's function solution for $\mu_a(\lambda_{BB}, t)$; $\mu_s'(\lambda_{BB}, t)$ was an input in this fit. Note, the labeled arrows connect the inputs for each step to the derived outputs.

In step 3, the wavelength-independent $A(t_{FD})$ and $b(t_{FD})$ were interpolated (linearly) to obtain $A(t)$ and $b(t)$ at the bDOS time points. Specifically, $A(t)$ and $b(t)$ were determined from $A(t_{FD})$and $b(t_{FD})$ and $A(t_{FD} + 30\text{ min})$and $b(t_{FD} + 30\text{ min})$ (see Figure 1, main text). The resultant $A(t)$ and $b(t)$ were then used to calculate the broadband tissue reduced scattering coefficient as a function of wavelength, *i.e.*, $\mu_s'(\lambda_{BB}, t) = A(t)(\lambda_{BB}/(500\text{ nm}))^{-b(t)}$ (step 4).

To compare the measured bDOS reflectance to theory, it is necessary to estimate a tissue coupling coefficient for the bDOS system, S (step 5). We do this using the FD-DOS measurement of

optical properties. $S(t)$ is defined as the multiplicative factor that converts the bDOS reflectance, $R(\lambda_{BB},t)$, to the "true" tissue diffuse reflectance spectra predicted by photon diffusion theory, $R_{\text{true}}(\lambda_{BB},t)$, *i.e.*, $R_{\text{true}}(\lambda_{BB},t) = S(t)R(\lambda_{BB},t)$. Note, here we assume that S does not depend on wavelength, but we permit it to change with time. To estimate S from FD-DOS measurements, we also assume that $R_{\text{true}}(\lambda_{BB},t)$ is well described by the semi-infinite continuous-wave photon diffusion Green's function solution.[4] Accordingly, we determine $R_{\text{true}}(\lambda_{FD},t_{FD})$ at the FD-DOS wavelengths and measurement times using the FD-DOS measurements of $\mu_a(\lambda_{FD},t_{FD})$ and $\mu_s'(\lambda_{FD},t)$ as inputs to the semi-infinite solution; additionally, the source-detector distance is set to 3 cm, an assumed wavelength-independent tissue refractive index is set to 1.4, and an assumed effective Fresnel reflection coefficient is set to 0.0065 (it was computed, as described elsewhere,[2] based on the refractive index of the optical probe being 1.45). Then, using this determination of $R_{\text{true}}(\lambda_{FD},t_{FD})$, we solve the following system of equations to estimate $S(t_{FD})$ via least-squares fitting (*lscov*, MATLAB):

$$\begin{pmatrix} R(\lambda_{FD,1},t_{FD}) \\ \vdots \\ R(\lambda_{FD,4},t_{FD}) \end{pmatrix} S(t_{FD}) = \begin{pmatrix} R_{\text{true}}(\lambda_{FD,1},t_{FD}) \\ \vdots \\ R_{\text{true}}(\lambda_{FD,2},t_{FD}) \end{pmatrix}. \quad \textbf{(1)}$$

In step 6, we simply linearly interpolate $S(t_{FD})$ between data taken at t_{FD} and $t_{FD} + 30\ \text{minutes}$ to all bDOS times, thereby giving $S(t)$. The resultant time-dependent multiplicative factor is then used in step 7 to calculate the true reflectance at all wavelengths, $R_{\text{true}}(\lambda_{BB},t) = S(t)R(\lambda_{BB},t)$. In step 8, the cerebral tissue absorption spectra, $\mu_a(\lambda_{BB},t)$, are obtained by fitting $R_{true}(\lambda_{BB},t)$ to the semi-infinite continuous-wave photon diffusion Green's function solution; $\mu_s'(\lambda_{BB},t)$ is used as an input for this fit. Note, a separate fit was performed for each wavelength, λ_{BB}, using the function *fminsearchbnd* ("fminsearchbnd, fminsearchcon - File Exchange - MATLAB Central," n.d.) with the constraint that $\mu_a(\lambda_{BB},t)$ must be between 0.001 and 1 cm^{-1}. Example measurements are shown in Figure S2.

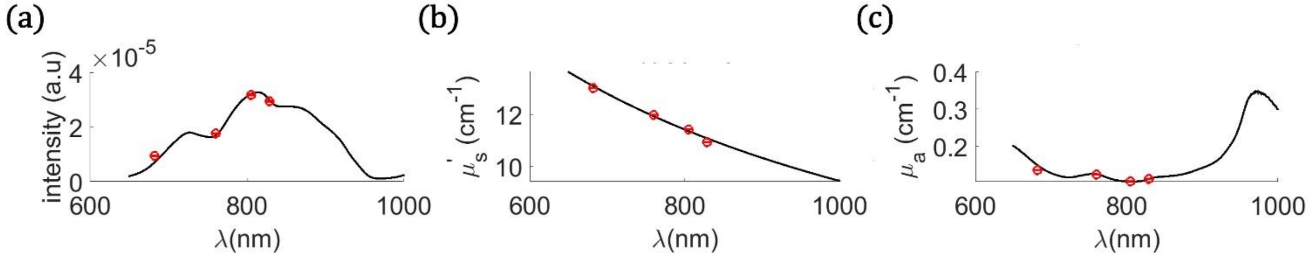

Figure S2: Example one-minute average of spectral measurements acquired on a pig during the study's baseline period. **(a)** Measurement of a "true" tissue diffuse reflectance spectrum ($R_{\text{true}}(\lambda)$, black curve) acquired with bDOS after FD-DOS calibration (the red circles denote the tissue diffuse reflectance spectra predicted by photon diffusion theory at the FD-DOS measured tissue optical properties). **(b)** FD-DOS measurements of the tissue reduced scattering coefficient (red circles) along with their fit (black curve) to a wavelength-dependent power law model for tissue scattering; the Mie scattering amplitude, A, and scattering power, b, are obtained from this fit. **(c)** Measurement of the tissue absorption spectrum (black curve) obtained from the conversion of $R_{\text{true}}(\lambda)$ (see Figure S1). The red circles denote the FD-DOS measured tissue absorption coefficients.

S.3 Optical Spectroscopy for Determination of OEF, ΔoxCCO, and HbT

We used the following relation to recover the tissue chromophore concentrations from the broadband absorption spectra ($\mu_a(\lambda_{BB},t)$):[4]

$$\mu_a(\lambda_{BB},t) = \sum_i \varepsilon_i(\lambda_{BB})c_i(t), \quad \textbf{(2)}$$

where $\varepsilon_i(\lambda_{BB})$ is the wavelength-dependent extinction coefficient and $c_i(t)$ is the concentration of the *i*th chromophore at time *t*. Note, Equation (2) is a system of equations; there is one equation for each light wavelength measured, *i.e.,* between 650 and 1000 nm. The tissue chromophores considered herein, all with known extinction coefficients, are oxy-hemoglobin (HbO_2), deoxy-hemoglobin (HbR), water, oxidized CCO (oxCCO), and reduced CCO (redCCO).

First, we took the time average of Equation (2) across the baseline period (see Figure 1, main text) and solved the resultant system of equations for the baseline HbO_2, HbR, and water concentrations. Note, due to the small effects of oxCCO and redCCO on the absorption spectrum, it was not feasible to recover absolute oxCCO and redCCO concentrations. Thus, using the same approximation typically employed by traditional near-infrared spectroscopy (NIRS) measurements,[4,5] we ignored the effects of oxCCO and redCCO in the solution to Equation (2) at baseline (*i.e.,* baseline oxCCO and redCCO concentrations were set to zero in Equation (2)).

The recovery of temporal changes in oxCCO and redCCO concentration with respect to baseline is tractable, if the total CCO concentration is assumed to remain constant.[6,7] Then, the differential change in the cerebral absorption spectra relative to baseline, *i.e.,* $\Delta\mu_a(\lambda_{BB},t) \equiv \mu_a(\lambda_{BB},t) - \langle\mu_a(\lambda_{BB})\rangle_{\text{Baseline}}$, is:[6]

$$\Delta\mu_a(\lambda_{BB},t) = \varepsilon_{\text{HbO}_2}(\lambda_{BB})\Delta HbO_2(t) + \varepsilon_{\text{HbR}}(\lambda_{BB})\Delta HbR(t) + \varepsilon_{\text{diffCCO}}(\lambda_{BB})\Delta oxCCO\,(t). \quad \textbf{(3)}$$

Here, $\Delta HbO_2\,(t)$, $\Delta HbR\,(t)$ and $\Delta oxCCO\,(t)$ are chromophore concentration changes relative to baseline, and $\varepsilon_{\text{diffCCO}}(\lambda_{BB}) = \varepsilon_{\text{oxCCO}}(\lambda_{BB}) - \varepsilon_{\text{redCCO}}(\lambda_{BB})$. We used the *in vivo* measurements of $\varepsilon_{\text{diffCCO}}(\lambda_{BB})$ published elsewhere ("UCL Medical Physics and Biomedical Engineering | Medical Physics and Biomedical Engineering - UCL – University College London," n.d.). Prior work has also suggested that the optimal wavelengths to use for deriving temporal oxCCO changes via Equation (3) is the 780-900 nm subset of the broadband wavelengths.[6,7] Thus, we solved the system of equations given by Equation (3) for the 780-900 nm subset of wavelengths to obtain the chromophore concentration changes.

To derive the absolute concentration of HbO_2 and HbR at each bDOS measurement timepoint *t*, the recovered changes in concentrations obtained from Equation (3) were added to the absolute concentrations at baseline obtained from Equation (2). Then the cerebral total hemoglobin concentration (HbT), cerebral tissue blood oxygen saturation (StO_2), and cerebral oxygen extraction fraction (OEF) were calculated:[2] $HbT(t) = HbO_2(t) + HbR(t)$, $StO_2(t) = HbO_2(t)/HbT(t)$, $OEF\,(t) = \big(SaO_2(t)\, -\, StO_2(t)\big)/\big(0.75 \times SaO_2(t)\big)$. Note, $SaO_2(t)$ in the OEF calculation is the arterial oxygen saturation obtained from linear interpolation of the arterial blood gas measurements.

S.4 Calculation of arterial blood oxygen content (CaO_2)

CaO_2 is readily derived from the arterial blood gas measurements:[8,9]

$$CaO_2 = \left(1.39\,\frac{\text{mL O}_2}{\text{g Hb}}\right) \times SaO_2 \times \left(32.2\,\frac{\text{g Hb}}{\text{dL blood}}\right) \times Hct + \left(0.003\,\frac{\text{mL O}_2}{\text{dL blood}\times\text{mmHg}}\right) \times PaO_2. \quad \textbf{(4)}$$

Here, SaO_2, PaO_2, and Hct denote the arterial blood gas measurements of arterial oxygen saturation (as a fraction, not a percentage), partial arterial oxygen pressure (in units of mmHg), and the hematocrit (as a fraction, not a percentage). Note, the second PaO_2 term in Equation (4) is negligible for the PaO_2 levels measured in this study.

S.5 Other Analysis Methods for the Determination of ΔoxCCO

Several different analysis methods have been used to quantify changes in oxCCO and hemoglobin with optical spectroscopy. These include: 1) a modified Beer-Lambert scheme for bDOS data using assumed differential pathlengths;[10-12] 2) a modified Beer-Lambert scheme for bDOS data using differential pathlengths estimated with either FD-DOS[13,14] or derivative spectroscopy[15,16] measurements; 3) semi-infinite photon diffusion theory fits to multispectral time-domain near-infrared spectroscopy data at 16 wavelengths,[17-19] and 4) semi-infinite photon diffusion theory fits to hybrid bDOS and FD-DOS or time-domain data.[20,21] In this contribution, we employed the 4th method.

The benefits of photon diffusion theory compared to the modified Beer-Lambert scheme include its higher accuracy for large chromophore concentration changes, and its ability to account for changes in tissue scattering.[18,20] Per the comparisons we mentioned in Section 4.3 of the main text, we used the 1st method (with assumed differential pathlength factor at 780 nm of 7.0) to analyze the data.

S.6 Routes of Hydrolysis of NV354 Succinate Prodrug to Succinate

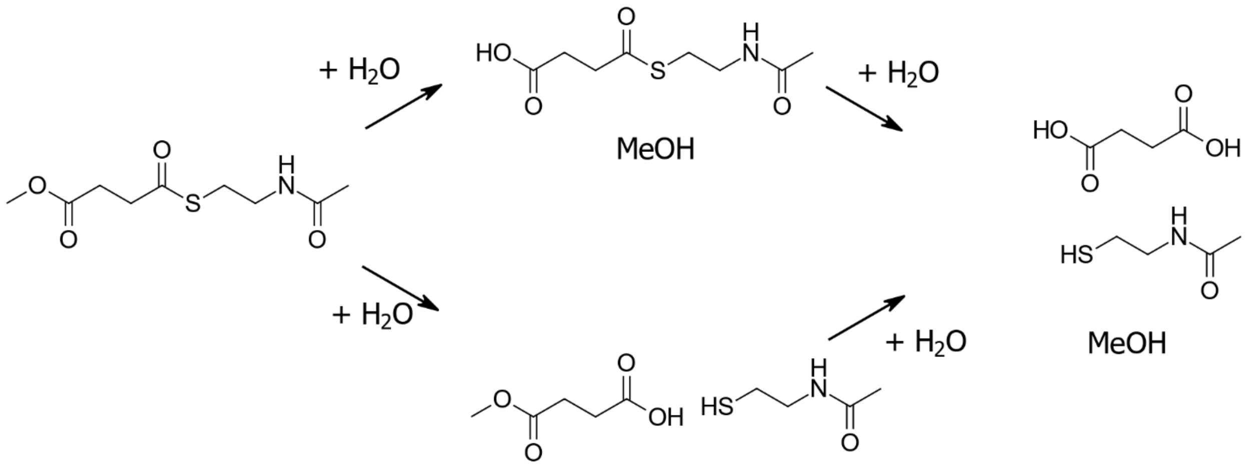

Figure S3: Schematic of the two potential routes of hydrolysis of the NV354 succinate prodrug (structural formula, left) once it is inside the cell. Both routes lead to succinate (structural formula, top right).

S.7 Magnetic Resonance Spectroscopy (MRS) measurements

Recall from the main text that after the 4-hour acute infusion period, the animals were transferred to a MRI scanner for MRS imaging of the following metabolites: myo-inositol, choline, N-acetylaspartate/creatinine, Glx (glutamate + glutamine), and lactate + lipid. Each metabolite was normalized to creatine. MRSI metabolites were computed from multiple gray-matter and white-matter regions (basal ganglia, cortex, hippocampus, thalamus, subcortical white matter, the genu, and selenium of corpus callosum). No differences in metabolites were found in any region, only basal ganglia are shown (Figure S4). Note, the temporality of metabolic changes that result in MRS signatures has not been well-studied, so the lack of significant difference in metabolites does not mean that there is no difference in neural damage between groups.

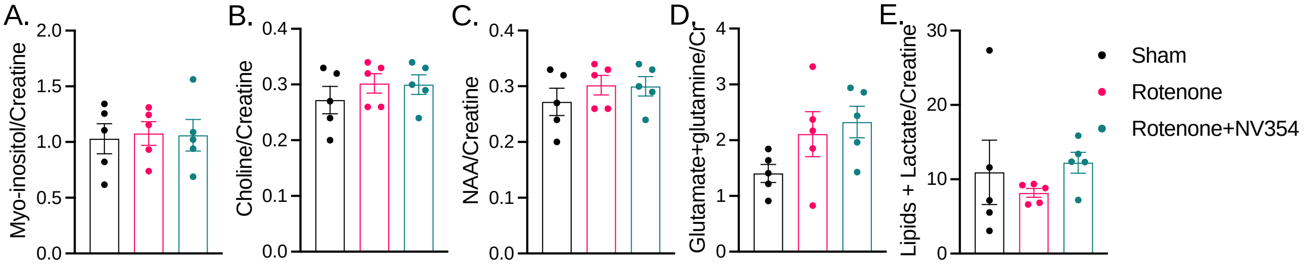

Figure S4. Metabolite ratios detected by magnetic resonance spectroscopy in the basal ganglia after 6h of rotenone infusion. No changes in myo-inositol (A), choline (B), N-acetylaspartate/creatinine (NAA; C), glutamate and glutamine (D) or lipids and lactate (E) to creatine ratios were found by MRS across 7 regions of interest (only basal ganglia depicted).

S.8 Supplementary Material References

1 Hueber, D. M. *et al.* Non-invasive and quantitative near-infrared haemoglobin spectrometry in the piglet brain during hypoxic stress, using a frequency-domain multidistance instrument. *Phys Med Biol* **46**, 41-62, doi:10.1088/0031-9155/46/1/304 (2001).

2 Forti, R. M. *et al.* Non-invasive diffuse optical monitoring of cerebral physiology in an adult swine-model of impact traumatic brain injury. *Biomed Opt Express* **14**, 2432-2448, doi:10.1364/boe.486363 (2023).

3 Bevilacqua, F., Berger, A. J., Cerussi, A. E., Jakubowski, D. & Tromberg, B. J. Broadband absorption spectroscopy in turbid media by combined frequency-domain and steady-state methods. *Appl Opt* **39**, 6498-6507, doi:10.1364/ao.39.006498 (2000).

4 Durduran, T., Choe, R., Baker, W. B. & Yodh, A. G. Diffuse Optics for Tissue Monitoring and Tomography. *Rep Prog Phys* **73**, doi:10.1088/0034-4885/73/7/076701 (2010).

5 Ayaz, H. *et al.* Optical imaging and spectroscopy for the study of the human brain: status report. *Neurophotonics* **9**, S24001, doi:10.1117/1.NPh.9.S2.S24001 (2022).

6 Bale, G., Elwell, C. E. & Tachtsidis, I. From Jöbsis to the present day: a review of clinical near-infrared spectroscopy measurements of cerebral cytochrome-c-oxidase. *J Biomed Opt* **21**, 091307, doi:10.1117/1.Jbo.21.9.091307 (2016).

7 Leadley, G., Austin, T. & Bale, G. Review of measurements and imaging of cytochrome-c-oxidase in humans using near-infrared spectroscopy: an update. *Biomed Opt Express* **15**, 162-184, doi:10.1364/boe.501915 (2024).

8 Benson, E. J. *et al.* Diffuse Optical Monitoring of Cerebral Hemodynamics and Oxygen Metabolism during and after Cardiopulmonary Bypass: Hematocrit Correction and Neurological Vulnerability. *Metabolites* **13**, doi:10.3390/metabo13111153 (2023).

9 Ko, T. S. *et al.* Non-invasive optical neuromonitoring of the temperature-dependence of cerebral oxygen metabolism during deep hypothermic cardiopulmonary bypass in neonatal swine. *J Cereb Blood Flow Metab* **40**, 187-203, doi:10.1177/0271678x18809828 (2020).

10 Bale, G. *et al.* Oxygen dependency of mitochondrial metabolism indicates outcome of newborn brain injury. *J Cereb Blood Flow Metab* **39**, 2035-2047, doi:10.1177/0271678x18777928 (2019).

11 Milej, D. *et al.* Assessing the relationship between the cerebral metabolic rate of oxygen and the oxidation state of cytochrome-c-oxidase. *Neurophotonics* **9**, 035001, doi:10.1117/1.NPh.9.3.035001 (2022).

12 Siddiqui, M. F. *et al.* Non-invasive measurement of a metabolic marker of infant brain function. *Sci Rep* **7**, 1330, doi:10.1038/s41598-017-01394-z (2017).

13 Saeed, F., Carter, C., Kolade, J., Brothers, R. M. & Liu, H. Understanding metabolic responses to forearm arterial occlusion measured with two-channel broadband near-infrared spectroscopy. *J Biomed Opt* **29**, 117001, doi:10.1117/1.Jbo.29.11.117001 (2024).

14 Wang, X., Tian, F., Soni, S. S., Gonzalez-Lima, F. & Liu, H. Interplay between up-regulation of cytochrome-c-oxidase and hemoglobin oxygenation induced by near-infrared laser. *Sci Rep* **6**, 30540, doi:10.1038/srep30540 (2016).

15 Matcher, S., Cope, M. & Delpy, D. Use of the water absorption spectrum to quantify tissue chromophore concentration changes in near-infrared spectroscopy. *Physics in Medicine & Biology* **39**, 177 (1994).

16 Rajaram, A. *et al.* Simultaneous monitoring of cerebral perfusion and cytochrome c oxidase by combining broadband near-infrared spectroscopy and diffuse correlation spectroscopy. *Biomed Opt Express* **9**, 2588-2603, doi:10.1364/boe.9.002588 (2018).

17 Lange, F., Dunne, L. & Tachtsidis, I. Evaluation of Haemoglobin and Cytochrome Responses During Forearm Ischaemia Using Multi-wavelength Time Domain NIRS. *Adv Exp Med Biol* **977**, 67-72, doi:10.1007/978-3-319-55231-6_10 (2017).

18 Lange, F., Dunne, L., Hale, L. & Tachtsidis, I. MAESTROS: A Multiwavelength Time-Domain NIRS System to Monitor Changes in Oxygenation and Oxidation State of Cytochrome-C-Oxidase. *IEEE J Sel Top Quantum Electron* **25**, 7100312, doi:10.1109/jstqe.2018.2833205 (2019).

19 Li, N. C., Ioussoufovitch, S. & Diop, M. HyperTRCSS: A hyperspectral time-resolved compressive sensing spectrometer for depth-sensitive monitoring of cytochrome-c-oxidase and blood oxygenation. *J Biomed Opt* **29**, 015002, doi:10.1117/1.Jbo.29.1.015002 (2024).

20 Lee, J., Armstrong, J., Kreuter, K., Tromberg, B. J. & Brenner, M. Non-invasive in vivo diffuse optical spectroscopy monitoring of cyanide poisoning in a rabbit model. *Physiol Meas* **28**, 1057-1066, doi:10.1088/0967-3334/28/9/007 (2007).

21 Eskandari, R. *et al.* Quantification of oxidized and reduced cytochrome-c-oxidase by combining discrete-wavelength time-resolved and broadband continuous-wave near-infrared spectroscopy. *Biomedical Optics Express* **16**, 3797-3812 (2025).
